# To treat or not to treat? Impact of hygienization on the microbiological safety of frass from black soldier fly larvae and yellow mealworm production aimed for fertilizer use

**DOI:** 10.1186/s40643-026-01030-7

**Published:** 2026-05-04

**Authors:** Ann De Volder, Lotte Frooninckx, David Deruytter, Johan Ceusters, Dries Vandeweyer

**Affiliations:** 1 Research Group for Insect Production and Processing (IP&P), Department of Microbial and Molecular Systems (M2S), KU Leuven, Geel Campus, Kleinhoefstraat 4, 2440 Geel, Belgium; 2 Research Group for Sustainable Crop Production and Protection (SusCroPP), Department of Biosystems, KU Leuven, Geel Campus, Kleinhoefstraat 4, 2440 Geel, Belgium; 3 Center of Expertise Sustainable Biomass and Chemistry, Thomas More University of Applied Sciences, Kleinhoefstraat 4, 2440 Geel, Belgium; 4 Insect Research Center Inagro, Ieperseweg 87, 8800 Rumbeke-Beitem, Belgium

**Keywords:** Insect rearing residue, *Hermetia illucens*, *Tenebrio molitor*, Hygienization, *Salmonella*, *E. coli*, Enterococaceae

## Abstract

**Abstract:**

Sustainable expansion of the insect farming industry requires valorization of the generated frass, a by-product with fertilizer and soil improver potential. Commercialization is currently restricted by Regulation (EU) No 2021/1925, considering insect frass as animal manure and imposing a pathogen-reducing heat treatment. This study investigated the microbiological safety of frass from industrial black soldier fly larvae (BSFL) and yellow mealworm (YM) production, untreated and hygienized by drying, pelleting or composting. Results showed that despite high microbial counts for both viable cells and aerobic endospores, *Salmonella* spp. were absent in untreated frass, *Escherichia coli* counts were low compared to other manure types, while the number of Enterococci was similar. The hygienisation procedures induced little change in the total number of viable cells and aerobic endospores and exerted more impact on temperature-sensitive Enterobacteriaceae compared to more tolerant Enterococcaceae. Drying, pelleting and composting were not equally suited for hygienization of frass from both insect species due to differences in initial microbial load and physical composition. Generally, it could be concluded that YM frass will meet the EU microbiological criteria more easily than BSFL frass and may even do so without hygienization. In case hygienization is necessary, reduction of the number of *E. coli* by the applied treatment will be the factor determining regulation compliance. Compared to Enterococcaceae, *E. coli* abundance in frass is lower and the species is more efficiently reduced. Oven drying is suited for both BSFL and YM frass but is expensive as it requires an external heat source. Pelleting seems applicable to YM frass, BSFL frass requires preliminary heating to reduce its moisture content. Composting of BSFL frass may produce a safe product if temperatures reached throughout the process are high enough. Composted YM frass can be considered microbiologically more unstable/unsafe than untreated YM frass, due to water addition at the process start. It may be concluded that flexibility in treatment procedures for insect frass, aimed for use as fertilizer, can be allowed. Microbiological safety is ensured after reference treatment (70 °C–60 min) but can also be achieved after milder processing or even without treatment, which would reduce costs and preserve frass quality.

**Graphical abstract:**

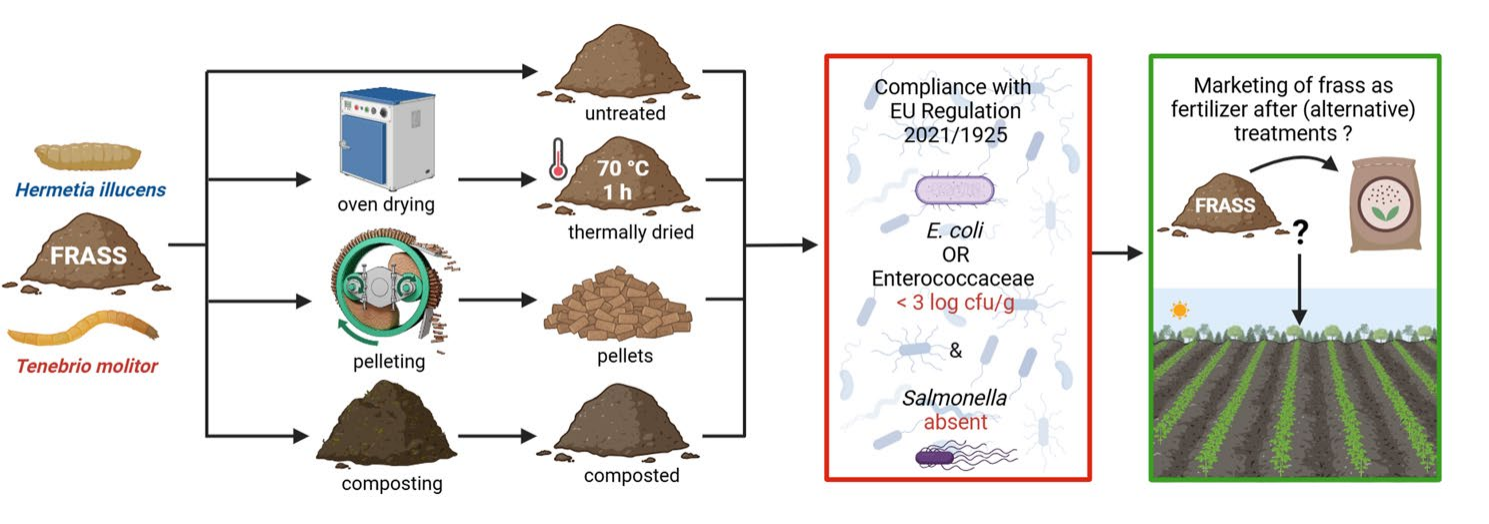

**Supplementary Information:**

The online version contains supplementary material available at 10.1186/s40643-026-01030-7.

## Introduction

Industrial insect farming has gained momentum in the past decade, driven by the rising demand for nutritious and sustainably produced feed and food. In Europe, black soldier fly larvae (*Hermetia illucens* L.), yellow mealworms (*Tenebrio molitor* L.) and house crickets (*Acheta domesticus* L.) are the most reared species (IPIFF 2021). The rearing process converts substrates (vegetable origin, not containing meat or fish) into valuable biomass (larvae, adult insects) which can be further processed into proteins and fats. Large-scale insect rearing, mainly aimed at producing ingredients for animal feed, is expected to grow exponentially from 10 000 metric tons in 2022 to 870 000 metric tons by 2030 (IPIFF 2023). This will also boost the volume of by-products generated, which should be valorized, to reduce production costs and enhance the sustainability and circularity of insect farming. Dead insects, shed exoskeletons and chitin may be used as biofuel, in cosmetics, animal/human medicine and agriculture (Chavez and Uchanski 2021). Frass, the major rearing by-product, is a mix of undigested feeding substrate, insect (parts), insect excrements, insect sheddings and dead eggs. Depending on its nature, 1000 kg of feeding substrate generates between 200 and 600 kg of frass (Lopes et al. 2022). European frass production may be more than 2.8 million metric tons by 2030 (IPIFF 2023). Several studies suggest that frass may have potential as soil amendment and bio-fertilizer in agriculture, resulting from its richness in nutrients and microorganisms (Poveda et al. 2019; Houben et al. 2020; Gärtling and Schulz 2021). However, some of these microorganisms such as the enterobacteria *Salmonella* and *Escherichia coli* (*E. coli*) as well as lactic acid bacteria from the family Enterococcaceae may pose health risks to consumers, given their potential entry into the food chain during application of frass in crop cultivation. In the European Union, placing on the market of insect frass as organic fertilizer and/or soil improver is currently restricted by Regulation (EU) No 2021/1925 (EC 2021), which defines frass and aligns standards for use with those applicable to processed animal manure. These standards imply that frass should be subjected to (1) a heat treatment process of at least 70 °C for at least 1 h and to (2) a reduction in spore-forming bacteria when they are identified as a relevant hazard. Representative samples taken during or immediately after processing must comply with the microbiological criteria shown in Table 2. The hygienization process itself, however, is not specified. Alternative methods are allowed given “it is demonstrated they ensure minimizing of biological risks” (EC 2011). The most frequently used treatments for solid animal manure, aimed for use as fertilizer, are physical thermal drying (dry heating, pelleting) and biothermal drying (composting) (Lemmens et al. 2017). Since July 2025, according to Regulation (EU) No 2025/1377, composted insect frass is allowed for marketing as fertilizer/soil improver on the same conditions as those applicable to heat-treated frass (EC 2025). Thermal drying is expensive as it requires an external heat source. In the composting process microorganisms generate heat while digesting organic matter. Both treatments reduce manure water content, resulting in 75 to 85% dry matter (dm) for thermally dried and 60 to 70% dm for composted manure (Melse and De Buisonjé 2020). Pellets are formed by extrusion under pressure, in this process, friction between solid particles and extruder walls generates heat. Since the pelleting process requires an input product with moisture content between 10 and 20%, manure pretreatment (drying) may be necessary. The produced cylindrical pellets are easier to store, transport and apply in agriculture compared to the loose dried/composted manure (Sharara et al. 2021).

To expand sustainable insect farming, valorization of the generated frass as fertilizer is a major factor. Next to killing living insects/insect eggs remaining in the frass after larvae harvesting, it is essential that the imposed hygienization treatment not only reduces the number of potential food pathogens in the frass, but also preserves its nutrient and microbiological quality, in an energy/cost-efficient manner, resulting in a marketable, economically competitive product. Evaluation of how the microbiological quality and safety of* H. illucens* (HI) and *T. molitor* (TM) frass was impacted by lab-scale thermal drying (water bath/closed containers) revealed differences in microbial load and response to heat treatment. Findings concerning compliance with EU microbiological safety criteria after less severe treatments (shorter time/lower temperature) opened perspectives on alternative hygienization methods and on preservation of potentially beneficial microorganisms (De Volder et al. 2025). However, given the applied lab-scale settings differ largely from frequently applied physical and biothermal industrial drying procedures, findings need further evaluation.

The aim of this study was to examine the impact of large scale hygienization methods on the microbiological quality and safety of frass from *H. illucens* and *T. molitor* rearing. General microbiological parameters as well as potential pathogens (*E. coli*, Enterococcaceae and *Salmonella*) were examined for untreated, thermally (oven) dried, pelletized and composted frass. Compliance with EU legislation for the marketing of frass as organic fertilizer/soil improver was assessed.

## Materials and methods

### Frass sources

Frass (untreated and dried) from the rearing of black soldier fly larvae and untreated frass from yellow mealworm production were supplied by industrial insect rearing companies in The Netherlands and Belgium respectively. An overview of the samples, the applied hygienization treatments (as described in 2.2) and the corresponding batch and sample codes is presented in Table 1. Batches 1 to 3 were sampled at three different time points, with a 6**-**months interval between samplings. Untreated frass was stored at 4 °C prior to microbiological analysis. It should be mentioned that HI pellets and HI1 compost were produced from the industrially dried frass. For all other treatments, the untreated frass was used as starting material. For the third sampling point, TM compost, as well as TM and HI pellets, were not available due to problems during the composting and pelleting process.

**Table 1:** Overview of frass types, treatments and corresponding batch and sample codes

Insect species	Treatment	Sample code		
		Batch 1	Batch 2	Batch 3
*Hermetia illucens*	Untreated	HI1.F	HI2.F	HI3.F
	Industrial drying (min. 70 °C–1 h)	HI1.D	HI2.D	HI3.D
	Thermal oven drying (70 °C–1 h)	HI1.TD	HI2.TD	HI3.TD
	Pelleting	HI1.P	HI2.P	*
	Composting	HI1.C	HI2.C	HI3.C

*Tenebrio molitor*	Untreated	TM1.F	TM2.F	TM3.F
	Thermal oven drying (70 °C–1 h)	TM1.TD	TM2.TD	TM3.TD
	Pelleting	TM1.P	TM2.P	*
	Composting	TM1.C	TM2.C	*

*No sample available; HI: *H. illucens*, TM: *T. molitor*

F: untreated, D: industrially dried, TD: thermally oven-dried, P: pelletized; C: composted

HI1 and HI2 pellets and HI1 compost were produced from industrially dried frass (HI.D)

### Microbiological safety criteria

Regulation (EU) No 2021/1925 standards for production and placing on the market of insect frass as an organic fertilizer and/or soil improver are shown in Table 2 (EC 2021).

**Table 2:** Microbiological standards applying to the placing on the EU market of insect frass as fertilizer and soil improver, Regulation (EU) No 2021/1925

Sampling	Microorganism	Sample number	Criteria		
			c	m	M
During or immediately after processing	*Escherichia coli*	5	5	0	1000
	OR				
	Enterococcaceae	5	5	0	1000

During or on withdrawal from storage	*Salmonella*	5	0	0	0
			Absence in 25 g		

c = number of samples for which the bacterial count in colony forming units per gram (cfu/g) may be between m and M

m = threshold value (lower limit) for the number of bacteria

M = maximum value (upper limit) for the number of bacteria, if the bacteria number for one sample exceeds M the batch is considered insufficiently processed, 1000 cfu/g = 3 log cfu/g

### Hygienization procedures

Applied hygienization procedures included industrial drying (D), thermal oven drying (TD), composting (C) and pelleting (P). Industrial thermal drying of the HI frass was performed by the insect rearing company, following certified hygienization procedures (minimally 70 °C –1 h). Untreated HI and TM frass were both submitted to pilot scale thermal oven drying. A three-centimeter-thick frass layer was spread evenly in an aluminum tray and heated in an oven (Memmert, Schwabach, Germany). Temperature was monitored by means of a USB data logger (EL-USB-TP-LCD, Lascar Electronics Ltd. Salsbury, UK) placed in the central part of the frass. When a temperature of 70 °C was reached, the frass was further heated for 60 min. Pelleting was performed by Samagro N.V (Leisele, Belgium). As the pelleting process requires a dry starting product, the industrially dried HI frass was used to produce pellets, while untreated TM frass was dry enough to be used as such. Heap composting was performed by Inagro (Rumbeke, Belgium) using approximately 5 metric tons of untreated TM frass or industrially dried (HI1.C) as well as untreated (HI2.C and HI3.C) HI frass. At the process start, frass moisture content was adjusted to 50% using rainwater, if necessary, to ensure equal optimal conditions for microbial activity. Two metric tons of wood chips were added to improve structure and oxygen penetration of the compost pile. Thirty heat sensors (LogTag® TRIX-8, LogTag Inc., North America) were placed at different positions in the pile to monitor temperature. The pile was covered with a semi-permeable blanket and turned over every two to three days to homogenize and aerate the material. Compost samples from the pile were taken after 21 days. This period should be sufficient for hygienization but will be too short to obtain mature compost.

### Intrinsic parameters (water activity, dry matter content and pH)

Frass water activity (a_w_) was determined in fivefold using a LabMaster a_w_ meter (Novasina, Lachen, Switzerland) until measured a_w_ and temperature (25 °C) were stable for at least 5 min. For some frass samples, dry matter content (%) (ISO 11465) and pH (ISO 10390) were determined by Bodemkundige Dienst van België (Heverlee, Belgium).

### Microbiological analysis

For determination of total viable count (TVC), the numbers of Enterobacteriaceae, *E. coli*, aerobic endospores and Enterococcaceae in frass, the ISO-standards assembled by Dijk et al. (2015) were followed. For each of five replicates, a primary dilution (1/10) in sterile peptone physiological salt solution (PPS, 0.1% peptone (Biokar Diagnostics, Beauvais, France), 0.85% NaCl) was prepared in a Stomacher® bag, using 5 g of frass. The solution was homogenized for 60 s with a Bagmixer® (Interscience, SaintNom, France) and used to prepare a tenfold dilution series in PSS. Next, 1 ml of each dilution was pour-plated in twofold using the appropriate culture medium. TVC was determined on Plate Count Agar (PCA, Biokar Diagnostics) after incubation at 30 °C for 72 h. Enterobacteriaceae count was assessed on Violet Red Bile Glucose agar (VRBG, Biokar Diagnostics) after incubation at 37 °C for 24 h. Enterococcaceae count, more specific enterococci count, was determined on Kanamycin esculin Azide Agar (KAA, Millipore, Darmstadt, Germany) after 48 h at 37 °C and *E. coli* count on Tryptone bile-x Glucuronide Agar (TBX, VWR, Leuven, Belgium) incubated at 44 °C for 24 h. To enumerate aerobic endospores, the primary dilution was subjected to a heat shock (80 °C for 10 min) to kill the vegetative cells and activate the endospores, prior to preparation of the dilution series. The aerobic endospores were then counted after incubation on PCA for 24 h at 37 °C. Presence/absence of *Salmonella* in 25 g frass was assessed following the RAPID’*Salmonella* short protocol (Bio-Rad Laboratories, Temse, Belgium). To 25 g frass and 225 ml buffered peptone water, one selective RAPID’*Salmonella* capsule was added. This tenfold sample dilution was incubated for selective enrichment at 41.5 °C for 16 to 22 h. Subsequently, 10 µl of the enriched dilution was evenly spread in twofold on RAPID’*Salmonella* agar and incubated at 37 °C for 48 h.

### Statistical analysis

All statistical analyses were performed using JMP Pro 17.0.0 software package from SAS, considering a significance level of 0.05. In case normality (evaluated with Shaprio-Wilk test) and homoscedasticity (evaluated with O’Brien) were confirmed, one-way ANOVA followed by Tukey HSD post-hoc test was used to compare intrinsic parameters as well as microbial plate counts, between frass samples and between samples before and after heat treatment. In case data were normal but not homoscedastic (unequal variances), Welch's ANOVA with Steel–Dwass all pairs post-hoc test was used. The non-parametric Kruskal–Wallis test with a pairwise Wilcoxon test was used to compare data that did not meet the assumptions of normality.

## Results and discussion

### Intrinsic parameters

Analysis results for dry matter content and pH (single results) as well as a_w_ values (determined in fivefold) are presented in Table 3. Dry matter content for untreated HI frass ranged from 45 to 57% and was much lower than values between 84 and 89% for TM frass. Results are in accordance with previously reported values. Wynants et al. (2019) measured between 26 and 77% dm for the residue of black soldier fly larvae (BSFL) rearing, Van Looveren et al. (2021) reported a value of 64% dm and De Volder et al. (2025) found values between 55 and 71% dm. In a study on yellow mealworm (YM) frass from four different insect rearers, dry matter contents between 81 and 90% were reported (OVAM 2022), Praeg & Klammsteiner (2024) found a similar value of 88% dm and De Volder et al. (2025) reported dm contents between 83 and 89%. The difference in moisture content between BSFL and YM frass is inherent to the rearing process of both insect species. Yellow mealworms are grown primarily on dry feed (e.g. wheat bran, brewers spent grain) supplemented with small amounts of wet feed (e.g. carrots, chicory, apples) as moisture source. They are harvested after 8 to 11 weeks. Black soldier fly larvae develop in a period of 7 to 14 days in a substrate initially containing up to 70% water and the short lifespan limits water loss through evaporation and insect uptake (Coudron and Deleu 2023).

**Table 3:** Intrinsic parameters for untreated and hygienized *H. illucens* and *T. molitor* frass samples. % dm and pH values are single results, a_w_ values are mean and standard deviation of 5 replicates

Frass treatment		Sample	dm (%)	a_w_ (–)	pH (–)
Untreated		HI1.F	57.2	0.95 ± 0.00	7.8
		HI2.F	55.4	0.96 ± 0.00	7.5
		HI3.F	45.2	0.97 ± 0.00	7.8
Industrially dried		HI1.D	80.6	0.67 ± 0.00	5.4
		HI2.D	85.6	0.66 ± 0.00	6.0
		HI3.D	84.4	0.64 ± 0.01	6.6
Thermally oven-dried		HI1.TD	93.5	0.95 ± 0.00	*
(70 °C–60 min)		HI2.TD	*	0.98 ± 0.00	*
		HI3.TD	*	0.95 ± 0.00	*
Pelletized		HI1.P	86.0	0.62 ± 0.00	*
		HI2.P	85.0	0.62 ± 0.00	*
Composted		HI1.C	70.2	0.96 ± 0.00	8.9
		HI2.C	55.5	0.96 ± 0.00	7.4
		HI3.C	*	0.97 ± 0.00	*

Untreated		TM1.F	88.0	0.56 ± 0.01^b^	6.3
		TM2.F	84.0	0.83 ± 0.02^a^	6.8
		TM3.F	89.0	0.58 ± 0.06^b^	6.1
Thermally oven-dried		TM1.TD	95.0	0.27 ± 0.01^b^	*
(70 °C–60 min)		TM2.TD	*	0.55 ± 0.00^a^	*
		TM3.TD	*	0.24 ± 0.00^c^	*
Pelletized		TM1.P	88.0	0.64 ± 0.00^b^	*
		TM2.P	86.8	0.70 ± 0.00^a^	*
Composted		TM1.C	67.1	0.96 ± 0.00^a^	7.8
		TM2.C	53.2	0.96 ± 0.00^a^	7.6

dm: dry matter, a_w_: water activity; HI: *H. illuce*ns, TM: T. molitor, * not provided; 1,2,3: batch number

F: untreated, D: industrially dried, TD: thermally oven-dried, P: pelletized, C: composted

HI1.C was made from HI1.D; HI1.P and HI2.P were made from HI1.D and HI2.D respectively

a–c: a_w_ results for HI and TM frass respectively, per treatment, with the same letter in superscript do not differ significantly (*p* ≥ 0.05); no letter indicates no significant differences between treatments

Both industrial and oven drying increased dry matter content of HI frass by respectively 60 and 80% on average compared to the value for the untreated frass. Since the untreated TM frass was already relatively dry (87% dm on average), thermal oven drying increased this value by only 8%. Pelleting had little influence on % dm, changes were less than 7% compared to the start product values. Adjustment of frass moisture content to approximately 50% with rainwater at the start of the composting process, resulted in a final product containing 1.5 (HI1.C), 2.7 (TM1.C) and 2.9 (TM2.C) times more moisture than the respective start materials (HI1.D, TM1.F and TM2.F).

The average water activity measured for untreated HI frass was 0.96 ± 0.01 while the average TM frass value of 0.66 ± 0.13 was significantly lower. Untreated HI frass showed a limited a_w_ range (0.95 to 0.97), with no statistical difference between tested samples. For untreated TM frass, TMF.2 had a statistically higher a_w_ value (0.83) compared to both other samples, with values of 0.56 and 0.58, for TMF.1 and TMF.3, respectively. This difference was unexpected given feeding substrates and growth conditions were probably identical. Measured a_w_ values for HI frass are in accordance with previously reported water activities between 0.83 and 0.98 by Wynants et al. (2019) and Van Looveren et al. (2021, 2022). The lower a_w_ value found for YM frass is in accordance with the value of 0.57 reported by Cesaro et al. (2022) and values between 0.60 and 0.80 found by De Volder et al. (2025).

The industrial drying process reduced the high HI frass a_w_ value significantly to 0.66 ± 0.01. Lab-scale oven drying however had little effect on HI frass water activity while the already low TM frass a_w_ value was further reduced by 0.30 on average. The observed reduction was similar for all three samples, independent of their initial a_w_ value. Pelleting of industrially dried HI frass had no influence on its water activity. For TM frass on the other hand, water activity was raised (TM1.P) or reduced (TM2.P) by approximately 0.1 compared to the starting values of the untreated frass, 0.56 and 0.83 respectively. Composting of industrially dried HI frass (HI1.C) and untreated TM frass (TM1.C and TM2.C) significantly raised water activity values by 0.3 on average, resulting from water addition at the start of the composting process. Composting of untreated HI frass (HI2.C and HI3.C) did not influence the a_w_ value, the untreated frass contained sufficient moisture, no rainwater was added for composting.

Moisture content represents the total amount of water present in a product while the a_w_ value (between 0 and 1) is a measure for the water available for microbial growth and chemical reactions. In a low a_w_ environment (a_w_ ≤ 0.85) most microorganisms become dormant and at a_w_ below 0.60 no microbial proliferation can occur (Rolfe and Daryaei 2020). Microbial growth will therefore be possible in untreated HI frass but will be restricted or inhibited in untreated TM frass. With an a_w_ value only slightly above the growth limit of 0.60, it can be considered as microbiologically stable as industrially dried HI frass.

While oven drying reduces moisture content in both frass types, the effect on water activity is diverse and may result from differences in frass composition, inherent to the insect species and the rearing methods. Several studies mention that product physical structure (fine/coarse material) and chemical composition (water, carbon, fat, sugar content) influence water activity changes during thermal treatment (Biswas et al. 2019; Rolfe and Daryaei 2020; Liu et al. 2022). For relatively wet HI frass, oven heating at 70 °C for one hour reduces moisture content through evaporation of water, but the process time/temperature may not be sufficient to lower the water activity to values of a microbiologically stable product. For dry TM frass with an already low a_w_ value, a larger part of the heating energy supplied may be used to remove bound water. Given no information was supplied concerning the conditions of the industrial drying process and the pelleting processes, attempts to explain observed changes in intrinsic parameter values would be based on assumptions rather than on correct data. The measured intrinsic parameter values will be primarily used to explain possible observed differences in the effect of the applied treatments on the microbiological counts.

With an average pH value of 6.4 ± 0.4, untreated TM frass was slightly more acidic than untreated HI frass with a pH value of 7.7 ± 0.2. For YM and BSFL frass from commercial insect farming, Praeg & Klammsteiner (2024) reported similar pH values of 6.4 and 7.7, respectively. The more acidic nature of YM frass (pH 6.1 and 6.4) was also reported by Amorim et al. (2024), while in a study about frass from commercial rearers in four EU countries, pH values between 7.2 and 8.8 for BSFL frass were found (SUSINCHAIN 2022). The values are comparable with those of other organic fertilizers (pH 6–8) (Siddiqui et al. 2024). Microbial growth is optimal at pH 7, possible in a pH range between 5 and 9 and limited below pH 6 (Balamurugan et al. 2014). Growth of most species will be possible in untreated HI frass but may be less abundant in untreated TM frass.

### Microbiological analysis

To assess the microbiological quality and safety of HI and TM frass, general parameters (TVC, numbers of Enterobacteriaceae and aerobic endospores) as well as counts of potential pathogens mentioned in EU legislation (*E. coli*, Enterococci and *Salmonella*) were determined in fivefold for untreated frass and frass hygienized by drying, pelleting and composting.

#### General microbiological parameters

**Fig. 1:**
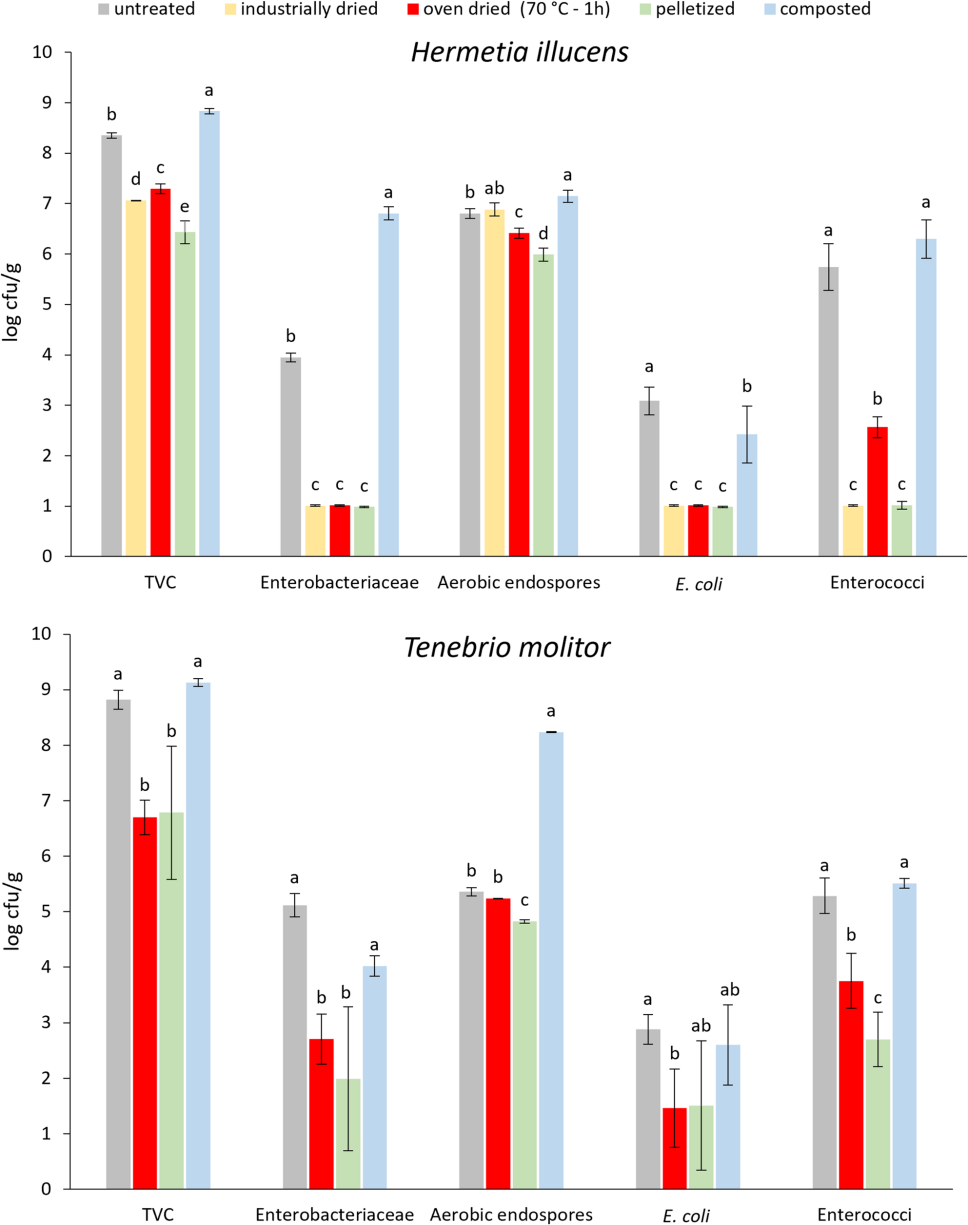
Comparison of TVC (total viable count), Enterobacteriaceae, aerobic endospore, *E. coli* and Enterococci counts (log cfu/g) between applied treatments (untreated (grey), industrially dried (yellow), oven dried (red), pelletized (green) and composted (blue)) for batch 2 frass samples from *H. illucens* (top) and *T. molitor* rearing (bottom). Results represent mean (bars) and standard deviation (error bars) of 5 replicates. a–e: mean of samples, per frass type, per bacterial species, with the same letter, do not differ significantly (*p* ≥ 0.05). Detection limit 1.0 log cfu/g. Legal limit for *E. coli* and Enterococcaceae: 3.0 log cfu/g

Analysis results for TVC, the numbers of Enterobacteriaceae and aerobic endospores in samples of untreated and hygienized frass from black soldier fly larvae and yellow mealworm rearing of batches 1, 2 and 3 are presented in Table 4. Comparison between treatments for the second frass batch is shown in Fig. 1.

**Table 4:** Results for TVC, Enterobacteriaceae and aerobic endospore counts for untreated and hygienized *H. illucens* and *T. molitor* frass samples

Frass treatment	Sample	Bacterial count (log cfu/g)		
		TVC	Enterobacteriaceae	Aerobic endospores
Untreated	HI1. F	8.0 ± 0.1^c^	5.3 ± 0.1^b^	6.2 ± 0.2^b^
	HI2. F	8.4 ± 0.0^b^	4.0 ± 0.1^c^	6.8 ± 0.1^a^
	HI3. F	8.8 ± 0.3^a^	6.0 ± 0.1^a^	6.1 ± 0.0^b^
Industrially dried	HI1.D	7.2 ± 0.7^a^	< 1.0 ± 0.0^a^	*
	HI2.D	7.1 ± 0.0^a^	< 1.0 ± 0.0^a^	6.9 ± 0.1^a^
	HI3.D	7.7 ± 0.2^a^	< 1.0 ± 0.0^a^	7.2 ± 0.1^a^
Thermally oven-dried (70 °C–60 min)	HI1.TD	6.9 ± 0.1^b^	< 1.0 ± 0.0^a^	6.2 ± 0.1^a^
	HI2.TD	7.3 ± 0.1^a^	< 1.0 ± 0.0^a^	6.4 ± 0.1^a^
	HI3.TD	6.9 ± 0.2^b^	1.1 ± 0.2^a^	5.5 ± 0.2^b^
Pelletized	HI1.P	7.8 ± 0.0^a^	1.5 ± 0.7^a^	7.2 ± 0.1^a^
	HI2.P	6.4 ± 0.2^b^	< 1.0 ± 0.0^b^	6.0 ± 0.1^b^
Composted	HI1.C**	9.5 ± 0.1^b^	< 1.0 ± 0.0^c^	7.3 ± 0.1^b^
	HI2.C	8.8 ± 0.1^c^	6.8 ± 0.1^b^	7.1 ± 0.1^b^
	HI3.C	10.1 ± 0.0^a^	7.2 ± 0.2^a^	8.2 ± 0.1^a^

Untreated	TM1. F	8.3 ± 0.1^b^	5.9 ± 0.2^a^	3.5 ± 0.2^b^
	TM2. F	8.8 ± 0.2^a^	5.1 ± 0.2^b^	5.4 ± 0.1^a^
	TM3. F	8.1 ± 0.4^b^	5.1 ± 0.1^b^	6.1 ± 1.0^a^
Thermally oven-dried (70 °C–60 min)	TM1.TD	7.1 ± 0.1^a^	4.9 ± 0.2^a^	3.7 ± 0.0^b^
	TM2.TD	6.7 ± 0.3^b^	2.7 ± 0.4^c^	5.2 ± 0.0^a^
	TM3.TD	6.5 ± 0.1^b^	4.0 ± 0.1^b^	4.0 ± 0.3^b^
Pelletized	TM1.P	7.3 ± 0.1^a^	2.3 ± 1.2^a^	6.1 ± 0.1^a^
	TM2.P	6.8 ± 1.2^a^	2.0 ± 1.3^a^	4.8 ± 0.0^b^
Composted	TM1.C	10.3 ± 0.2^a^	6.4 ± 0.3^a^	7.2 ± 0.1^b^
	TM2.C	9.1 ± 0.1^b^	4.0 ± 0.2^b^	8.2 ± 0.0^a^

Data are the mean value and standard deviation of five replicates; HI: *H. illucens,* TM*: T. molitor*

^*^Analysis not conducted; 1,2,3: batch number; ** HI1.C made from HI1.D

F: untreated, D: industrially dried, TD: thermally oven-dried, P: pelletized, C: composted

a–c: mean of samples, per frass type, per treatment, for TVC, Enterobacteriaceae and aerobic endospores respectively, with the same letter in superscript, do not differ significantly (*p* ≥ 0.05)

The untreated HI and TM frass had similar average TVC values of 8.4 ± 0.0 log cfu/g and comparable average Enterobacteriaceae counts (5.1 ± 0.9 and 5.3 ± 0.3 log cfu/g for HI and TM frass samples respectively). Untreated HI frass carried more aerobic endospores (6.3 ± 0.3 log cfu/g) than TM frass (4.9 ± 1.3 log cfu/g). Statistical differences were found between frass samples from the same insect in the 3 sampling batches but were not consistent for the 3 measured parameters. TVC values in untreated HI frass are slightly lower than values between 8.5 and 10.2 log cfu/g reported by Wynants et al. (2019) and Van Looveren et al. (2022). They also found higher Enterobacteriaceae counts, respectively between 5.7 and 9.5 log cfu/g and reported counts for aerobic endospores between 4.2 and 7.0 log cfu/g. TVC values for the untreated TM frass are considerably higher than those reported by Praeg & Klammsteiner, (2024) and Amorim et al. (2024) of respectively 5.9, 6.2 and 3.9 log cfu/g, but similar to counts between 7.9 log cfu/g and 8.7 log cfu/g found by De Volder et al. (2025). Enterobacteriaceae counts are in accordance with values between 4.7 log cfu/g and 6.5 log cfu/g reported in a study on eight YM frass samples from six insect rearers (OVAM 2022), while Osimani et al. (2018) reported slightly higher values (6.8 and 7.0 log cfu/g) and Amorim et al. (2024) found lower values (2.9 and 3.3 log cfu/g). Findings indicate considerable variation in microbiological load, both for frass from the rearing of different insect species and for different batches of the same species.

TVC values between 6 and 8 log cfu/g are considered normal for fresh raw vegetables and will increase rapidly upon decay (UK Health Security Agency 2024). As black soldier fly larvae are grown in a substrate consisting of vegetable waste streams, in a relatively humid and warm environment favoring microbial growth (Klammsteiner et al. 2020), a high microbial load of the residual frass is to be expected. Moreover, microorganisms belonging to the insect gut microbiota, such as Enterobacteriaceae, are also transferred to the substrate via excrements (De Smet et al. 2018). The microbial load of untreated HI and TM frass can be considered similar or slightly lower compared to other types of animal manure aimed for fertilizer use, such as cow and horse manure, pig and chicken litter, for which TVC and Enterobacteriaceae values from 8 to more than 10 log cfu/g are reported (Tontti et al. 2011; Chen and Jiang 2017).

Hygienization by drying minimally influenced TVC. Average changes were less than 2 log units, reduction was sometimes statistically significant, but microbiologically less relevant. In studies on the impact of heat treatment (70 °C–60 min) on BSFL frass, limited average TVC reduction (0.8 and 0.5 log units respectively) was also reported by Van Looveren et al. (2022). Praeg & Klammsteiner, (2024) reported a slight decrease in TVC for BSFL frass but none for YM frass. For aerobic endospores, reflecting the fraction of TVC present as spore forms, counts before and after oven drying of HI and TM frass were comparable, as could be expected given the heat resistance of bacterial endospores (Vandeweyer et al. 2020).

Drying significantly reduced the number of Enterobacteriaceae in HI frass, by 3 to 4 log units, resulting in an average count of 1.1 ± 0.2 log cfu/g. For 2 replicates of sample HI3.TD, reduction below the detection limit of 1.0 log cfu/g was not achieved, counts after oven drying at 70 °C for 1 h were 1.3 log cfu/g. In contrast, Enterobacteriaceae count reduction in TM frass was only 1.5 log units, with a residual average value of 3.9 ± 0.1 log cfu/g after oven drying. For BSFL frass, Van Looveren et al. 2022 reported a comparable reduction of the initial Enterobacteriaceae count of 7.1 log cfu/g to a value below detection limit, after heat treatment at 70 °C for 1 h. The only comparison data for YM frass were found in De Volder et al. (2025). They reported initial Enterobacteriaceae counts of 5.8 and 3.5 log cfu/g for two different YM frass samples. After heat treatment at 70 °C for 1 h, counts in the first sample were reduced to 1.8 log cfu/g but reduction below detection limit required a minimum temperature of 80 °C. For the second YM frass sample, with Enterobacteriaceae counts 2 log units lower, treatment at 70 °C during both 60 and 30 min reduced values below detection. Enterobacteriaceae are known to be sensitive to heat treatment, stronger reduction in BSFL frass is most likely related to its higher moisture content and water activity, which improve the efficacy of the heating process.

Changes in microbial counts after pelleting of the industrially dried HI frass were less than 1 log unit. Pelleting of untreated TM frass reduced TVC counts by less than 2 log units. In contrast, Enterobacteriaceae counts (5–6 log cfu/g) were significantly reduced to values around 2 log cfu/g, while the aerobic endospores counts either slightly increased (TM1.P) or remained unchanged (TM2.P). Pelleting requires a starting product with less than 20% moisture; pre-drying may therefore be necessary but raises processing costs. During pelleting the material is molded into cylindrical pellets of about 1 to 5 cm length, by means of compression or extrusion. The shear forces generate a temperature increase by 20 to 30 °C, additional injection of process steam may raise it far more. The elevated temperature negatively impacts bacterial survival, resulting in pellets with reduced pathogen count (Lemmens et al. 2006). In a study on chicken feed pellets, produced after steam conditioning at low (57 °C), medium (75 °C) and high (85 °C) temperature, followed by pelleting, Cox et al. (1986) also reported minimal reduction in TVC values. Influence on Enterobacteriaceae numbers (initial count 3.5 and 4.5 log cfu/g) during the conditioning process ranged from no reduction at low temperature to a maximum 1.5 log unit at the highest temperature. Further reduction after pelleting ranged from 1.7 to more than 3.5 log units and was most outspoken for the starting product containing less fat and more limestone, both factors generating more friction/higher temperatures during pelleting. Pelleting of chicken broiler feed inoculated with *Enterococcus faecium* at 5 log cfu/g resulted in a 1.5 log unit reduction at 70 °C after 15 s processing time, which increased to 2.5 log unit reduction at 80 °C for 75 s (Boltz et al. 2019). The temperature generated in the pelleting process applied in the current study is unknown, but it was sufficient to reduce Enterobacteriaceae counts in TM frass by more than 3 log units, which is twice the reduction observed after oven drying. As the samples did not differ in physical composition, it may be assumed that the temperatures generated during pelleting were higher than during oven drying. Increase in the number of aerobic endospores after pelleting may possibly result from contamination in the equipment used, originating from previously pelleted products.

Heap composting of untreated HI and TM frass increased TVC counts by maximum 2 log units. Aerobic endospore counts increased more in composted TM frass (3.3 log units) compared to HI frass (1.2 log units). In contradiction, changes in the number of Enterobacteriaceae were smaller for TM frass. A minor 0.5 log unit increase was observed for the TM1.C sample while TM2.C even showed a 1.1 log unit decrease in Enterobacteriaceae number, HI frass samples on the other hand showed an average 2 log unit increase. The process of aerobic heap composting consists of four phases (mesophilic, thermophilic, cooling and maturation phase), characterized by temperature and microbiologically active microorganisms. During the first phase, mesophilic microorganisms (growth range 20 to 45 °C) grow fast on the easily available nutrients (sugars, amino acids and lipids). The heat generated by their metabolic activity raises the temperature in the pile, up to the point where they are suppressed and activity is taken over by thermophilic spore-forming bacteria, actinobacteria and fungi (growth range 50 to 70 °C). They break down proteins, fats and carbohydrates, which further raises temperature to 55–65 °C or even higher, if the pile is efficiently aerated by regular turning over. When carbon and nitrogen sources become scarcer, temperature drops to 40–45 °C and mesophilic organisms grow again during the curing and maturation phases which can take several weeks or even months. Factors influencing inactivation of potential pathogens during composting are, besides temperature reached in the thermophilic phase, microbial antagonism and nutrient competition, changes in pH and moisture content. Generally, Enterobacteriaceae are least likely to survive composting, followed by Enterococcaceae and finally spore-forming bacteria (Chen and Jiang 2014; Lepesteur 2022; Ho et al. 2022). Temperature monitoring during composting (supplementary file Figure S1) revealed that only for HI2.C a maximum temperature of 70 °C was reached in the composting pile for at least one hour. For HI3.C a maximum temperature just below 70 °C was registered while for TM frass composting temperatures remained below 60 °C. The observed increase in bacteria numbers after heap composting may result from the combined effects of minor reduction by insufficient process temperature and growth during curing. Since the compost pile was in a non-sterile environment and was manipulated regularly, new species, including some from the Enterobacteriaceae family may have been introduced. Differences in aeration in the compost pile may have promoted growth of facultatively anaerobic Enterobacteriaceae. Although heap composting of frass requires no additional energy input, addition of wood chips (to improve the compost pile structure) and regular turning (to improve oxygen penetration) augment processing costs. In industrial settings, composting is performed in concrete tunnels, using larger volumes, climate control, intense moistening and aeration to obtain adequate pathogen reduction (Lemmens et al. 2006).

The high TVC, Enterobacteriaceae and aerobic endospore counts after processing do not inherently imply a safety risk for the use of insect frass as fertilizer but suggest that several bacterial species withstand treatment, either as viable cells or as endospores. The latter are formed under stress conditions (high temperature, nutrient scarcity, desiccation) as a bacterial survival strategy to preserve genetic material. They are metabolically dormant, but able to germinate and multiply on return of more favorable conditions (Rolfe and Daryaei 2020). Persistence or even increase of aerobic endospores is most outspoken after composting. Several articles mention the presence of species with traits related to promotion of plant growth and/or biostimulant activity in the microbiota of untreated BSFL and YM frass (Poveda et al. 2019; Poveda 2021; Lopes et al. 2022; Barragán-Fonseca et al. 2022; Beesigamukama et al. 2023). Their potential presence/survival in treated frass is promising, expanding the frass valorization potential in sustainable agriculture from simply a fertilizer/soil improver to a biostimulant, thereby creating additional marketing opportunities.

#### Microbiological parameters related to EU legislation

Besides for general microbiological quality, samples were tested for compliance with the EU criteria for marketing insect frass as fertilizer and soil improver (Table 2). Analysis results for the numbers of *E. coli* and Enterococci and for presence/absence of *Salmonella* in samples of untreated and hygienized frass from black soldier fly larvae and yellow mealworm rearing of batches 1, 2 and 3 are presented in Table 5. Comparison between treatments for the second frass batch is shown in Fig. 1.

**Table 5:** Results for *E. coli* and Enterococci counts and presence/absence of *Salmonella* spp. (in 25 g sample) for untreated and hygienized *H. illucens* and *T. molitor* frass

Frass treatment	Sample	*Escherichia coli*		Enterococci		*Salmonella*	
		(log cfu/g)	x/5 ≥ 3	(log cfu/g)	x/5 ≥ 3	Presence/absence	x ND
Untreated	HI1. F	*	*	*	*	ND in 25 g	5
	HI2. F	3.1 ± 0.3^b^	2	5.7 ± 0.5^b^	5	ND in 25 g	5
	HI3. F	4.6 ± 0.2^a^	5	6.4 ± 0.4^a^	5	ND in 25 g	5
Industrially dried	HI1.D	< 1.0 ± 0.0^a^	0	*	*	ND in 25 g	5
	HI2.D	< 1.0 ± 0.0^a^	0	< 1.0 ± 0.0^a^	0	ND in 25 g	5
	HI3.D	< 1.0 ± 0.0^a^	0	< 1.0 ± 0.0^a^	0	ND in 25 g	5
Thermally oven-dried (70 °C–60 min)	HI1.TD	< 1.0 ± 0.0^a^	0	*	*	ND in 25 g	5
	HI2.TD	< 1.0 ± 0.0^a^	0	2.6 ± 0.2^b^	0	ND in 25 g	5
	HI3.TD	< 1.0 ± 0.0^a^	0	4.8 ± 0.4^a^	5	ND in 25 g	5
Pelletized	HI1.P	< 1.0 ± 0.0^a^	0	1.9 ± 1.2^a^	0	ND in 25 g	5
	HI2.P	< 1.0 ± 0.0^a^	0	< 1.0 ± 0.1^a^	0	ND in 25 g	5
Composted	HI1.C	< 1.0 ± 0.0^a^	0	*	*	ND in 25 g	5
	HI2.C	2.4 ± 0.6^a^	1	6.3 ± 0.4^b^	5	ND in 25 g	5
	HI3.C	2.6 ± 0.2^a^	0	6.9 ± 0.2^a^	5	ND in 25 g	5

Untreated	TM1. F	*	*	*	*	ND in 25 g	5
	TM2. F	2.9 ± 0.3^a^	2	5.3 ± 0.3^b^	5	ND in 25 g	5
	TM3. F	< 1.0 ± 0.0^b^	0	6.5 ± 0.5^a^	5	ND in 25 g	5
Thermally oven-dried (70 °C–60 min)	TM1.TD	*	*	*	*	ND in 25 g	5
	TM2.TD	1.5 ± 0.7^a^	0	3.8 ± 0.5^b^	5	ND in 25 g	5
	TM3.TD	< 1.0 ± 0.0^a^	0	4.7 ± 0.4^a^	5	ND in 25 g	5
Pelletized	TM1.P	< 1.0 ± 0.0^a^	0	2.6 ± 0.4^a^	0	ND in 25 g	5
	TM2.P	1.5 ± 1.2^a^	1	2.7 ± 0.5^a^	2	ND in 25 g	5
Composted	TM1.C	*	*	*	*	ND in 25 g	5
	TM2.C	2.6 ± 0.7	1	5.5 ± 0.1	5	ND in 25 g	5

Data are the mean value and standard deviation of five replicates; *H. illucens* (HI), *T. molitor* (TM)

*Analysis not conducted; 1,2,3: batch number; ** HI1.C made from HI1.D

F: untreated; D: industrially dried; TD: thermally oven-dried; P: pelletized; C: composted

x/5 ≥ 3: number of replicates out of 5 with bacterial count ≥ 3 log cfu/g

a-b: mean of samples, per frass type, per treatment, for *E. coli* and Enterococci respectively, with the same letter in superscript, do not differ significantly (*p* ≥ 0.05)

*Salmonella* was not detected in all 5 replicates of all batches of untreated HI and TM frass. Hygienization, whether by drying, pelleting or composting, did not induce *Salmonella* contamination. Most studies on untreated BSFL and YM frass also mention that the species was not detected (Osimani et al. 2018, 2019; De Smet et al. 2021; OVAM 2022; Van Looveren et al. 2022). When inoculated in BSFL frass up to 5.3 log cfu/g, Van Looveren et al. (2022) observed that *Salmonella* spp. were no longer detected after heat treatment at 70 °C for 1 h (water bath, closed containers). In a similar experiment, De Volder et al. (2025) also observed efficient reduction of inoculated *Salmonella* spp. in both BSFL and YM frass. At lower temperature of 60° C the species was still detected in the YM frass after treatment.

Regulation (EU) No 2021/1925 specifies that presence/absence of *Salmonella* in frass should be tested on processed samples, taken during or on withdrawal from storage. It may be assumed that if *Salmonella* is not present in untreated or processed frass, growth during storage will not occur and external contamination can be avoided on application of proper hygiene standards. In the current study the time between treatment and analysis of the samples in the lab nor the exact storage conditions and times were consistent for all batches. Therefore, it will be necessary to conduct further studies on the behavior of (inoculated) *Salmonella* in insect frass after processing and subsequent storage under controlled conditions, to confirm these assumptions.

Average *E. coli* counts in untreated HI frass samples were significantly different, 3.1 ± 0.3 and 4.6 ± 0.2 log cfu/g for HI2.F and HI3.F, respectively. For the latter, counts of all replicates were above 3 log cfu/g. Although the species was not enumerated in HI1.F, considering the relation between Enterobacteriaceae and *E. coli* counts in both other samples, *E. coli* numbers around 4 log cfu/g may also be estimated for this sample. In untreated TM frass *E. coli* presence was lower, counts were below the detection limit in TM3.F and counts above 3 log cfu/g were only found in 2 out of 5 TM2.F replicates. On the other hand, untreated HI and TM frass showed similar average Enterococci counts of respectively 6.1 ± 0.5 and 5.9 ± 0.8 log cfu/g. In all replicates counts were above the legal limit of 3 log cfu/g. Amorim et al. (2024) reported a similar low *E. coli* concentration of 2.9 log cfu/g for YM frass while Swinscoe et al. (2019) found the species in minor numbers (1.2 log cfu/g) in one of two frass batches from BSFL raised on seaweed powder but reported comparable high Enterococci numbers (6 log cfu/g) for both batches. Other organic wastes used for fertilizer production also showed similar Enterococci numbers (4 to 7 log cfu/g for cow and swine manure) but higher *E. coli* counts: chicken litter (4–7 log cfu/g), cattle manure (6–7 log cfu/g) and swine manure (6 log cfu/g) (Lin et al. 2022). The lower prevalence of *E. coli* in frass from insect production may not only be related to lower occurrence of the pathogen in insect excrements but also to the pathogen reducing effect exerted by the insect larvae and the composition of the frass itself. Lopes et al. (2020) inoculated *E. coli* at 6 log cfu/g level in a mix of bread and aquaculture waste, used as substrate for BSFL larvae rearing. After two weeks the pathogen level was reduced to 2 log cfu/g while no reduction was observed in the inoculated control without larvae. Moreover, on re-inoculation of the substrate, the *E. coli* count of 5 log cfu/g was reduced by 2.5 log units when larvae were no longer present. Findings suggest pathogen reduction to be related to antimicrobial substances, secreted by the BSF larvae or originating from their associated microbial community. Van Looveren et al. (2024) investigated vertical transmission of *E. coli* across life stages of BSF through inoculation of approximately 7.0 log cfu/g of the pathogen in the rearing substrate. They also observed decreased *E. coli* numbers in the frass. Additionally, microorganisms ingested by the larvae may be unable to survive the low pH conditions (≤ 3) in the larval midgut, thereby contributing to a reduced abundance of viable bacteria in frass (Vandeweyer et al. 2023). In an experiment in which yellow mealworms were raised on wheat middlings, inoculated with *E. coli* at 6 log cfu/g, Cesaro et al. (2022) observed a reduction by 2 log units after only 2 weeks, and found the pathogen no longer detectable after 6 weeks of larval growth. As similar pathogen reduction was also observed in the inoculated substrate without larvae, both substrate and frass were considered environments unfavorable for *E. coli* survival, because of their low a_w_ value (0.58).

Industrial drying reduced *E. coli* and Enterococci numbers in HI frass below the detection limit. A similar *E. coli* count reduction was observed after oven drying at 70 °C for one hour. Reduction of Enterococci on the other hand was significant but lower; numbers below 3 log cfu/g were only achieved for the HI2.TD sample. The average count for sample HI3.TD was 4.8 ± 0.4 log cfu/g, with all 5 replicates above 3 log cfu/g. The industrial drying process conditions may have been more severe. The higher moisture content (55%, Table 3) of this frass sample may have influenced the oven drying process, as more of the heating energy supplied may have been used for water evaporation. For TM frass, despite a significant, average 1.7 log unit reduction after oven drying, Enterococci numbers for all replicates remained above the legal limit. As *E. coli* was not detected in untreated TM frass from the third batch, reduction could only be assessed for sample TM2. Before thermal oven drying, 2 out of 5 replicates showed *E. coli* counts ≥ 3 log cfu/g but after treatment all counts were below this limit, with counts for 3 out of 5 replicates even below the detection limit.

In a study on the effect of heat treatment (water bath, 70 °C–1 h) on cow manure, Delmon et al. (2021) observed a 1.4 log unit reduction of the initial *E. coli* count (3.4 log cfu/g) and a 2.7 log unit decrease for the number of *Enterococcus* sp. (6.8 log cfu/g), implying that counts below 3 log cfu/g were also only reached for *E. coli.* In a comparable study on solid pig slurry (67% dry matter), Pourcher et al. (2009) found that both *E. coli* and Enterococci were reduced to numbers below 3 log cfu/g, the initial concentrations for *E. coli* (4.4 log cfu/g) were higher while the number of Enteroccoci (4.8 log cfu/g) was lower. One-hour treatments at lower temperatures (55 and 60 °C) induced similar *E. coli* count reductions, but lower decreases in the number of Enterococci. When assessing the impact of heat treatment (water-bath, closed containers) on BSFL frass, De Volder et al. (2025), observed *E. coli* counts reduced to values below the detection limit of 1.0 log cf/g and Enterococci counts below 3 log cfu/g. For YM frass however, Enterococci reduction was lower as comparable initial counts of 7.8 log cfu/g were only reduced to counts between 4 and 5 log cfu/g. These findings confirm the observed higher resistance to heat treatment for Enterococci versus *E. coli* in HI and TM frass.

Composting of untreated HI frass reduced *E. coli* counts below 3 log cfu/g in all replicates of HI3.C and in all but one replicate of HI2.C while Enterococci numbers showed a slight increase and remained above the legal limit in all replicates of both samples. Composting of untreated TM frass sample TM2 induced no significant change in either *E. coli* or Enterococci number. Only one of the 5 replicates had an *E. coli* count above 3 log cfu/g after composting while Enterococci counts remained above this limit in all replicates, resulting in an average count of 5.5 ± 0.1 log cfu/g. In comparison, Miller et al. (2013) found no *E. coli* in plant and green waste compost, similar average low concentrations (2.5 log cfu/g) in composted horse manure but higher values (3.6 log cfu/g) for cow manure compost. Tontti et al. (2011) reported Enterococci numbers of 4.5 and 5.2 log cfu/g in two piles of heap-composted cattle manure, after 6 months of processing.

As mentioned earlier, the pathogen-reducing effect of composting relies strongly on temperature. The composting temperatures reached were probably inadequate for eradication of *E. coli* below detection limit in both HI and TM frass. The legal limit of 3 log cfu/g, however, was achieved in all but one replicate of both HI and TM frass. Composting had no reducing effect on Enterococci prevalence in insect frass.

*E. coli* and Enterococci are commonly used in health risk assessment as interchangeable indicator organisms for fecal contamination in the environment. Both are facultative anaerobic bacteria, commensals of the gastro-intestinal tracts of humans and animals (including insects) and excreted with their feces (Manyi-Loh et al. 2016; Li et al. 2021). Most *E. coli* strains are harmless, but some are pathogenic and may cause gastro-intestinal infections, meningitis and sceptic shock in humans, such as *E. coli* O157:H7 which is mostly found in cattle manure. By production of toxins and other virulence factors the strain can survive and damage cells in parts of the body where *E. coli* normally do not reside. Enterococci do not produce toxins but some species like *Enterococcus faecalis* and *E. faecium* may cause foodborne illness or healthcare-associated infections and have become increasingly antibiotic resistant (Byappanahalli et al. 2012; Li et al. 2021). Survival and proliferation of *E. coli* in the non-enteric environment is relatively poor but not impossible. Ongeng et al. (2015) reported that survival of *E. coli* O157:H7 in the manure-amended soil–plant ecosystem is favored by acid pH, low salinity, low temperature, adequate nutrient availability and weak competition of the soil/plant microbiome. Enterococci persistence is more robust as they can withstand a wide temperature range (10 to 45 °C), survive desiccation, high salinity and alkaline pH (up to pH 10) (Byappanahalli et al. 2012; Hubbart et al. 2022).

Based on the experimental findings it may be questionable whether *E. coli* and Enterococcaceae are equally suited to assess microbiological safety of (un)processed insect frass. The first discrepancy is the variation in their occurrence/numbers in frass samples from rearing batches of both the same and different insect species. The prevalence of *E. coli* seems to be higher in HI frass (1 up to 4.5 log cfu/g more) compared to TM frass, while Enterococci presence is similar (ca. 6 log cfu/g). Differences between rearing batches of the same insect species are more outspoken for TM frass (*E. coli*, 3 log unit difference, Enterococci < 1 log unit) compared to HI frass (both *E. coli* and Enterococci < 1 log unit difference). The second discrepancy is their different resistance to stresses. Enterococcaceae are generally more resilient to higher temperature and to lower moisture availability compared to *E. coli*. It may therefore be assumed and was also confirmed by conducted experiments, that heat treatment, whether by (oven) drying, pelleting or composting, has a larger reducing effect on the latter. Combined with a higher Enterococcaceae prevalence in insect frass it seems probable that, upon testing frass samples, *E. coli* numbers will meet the legal criteria more often and more easily.

**Fig. 2:**
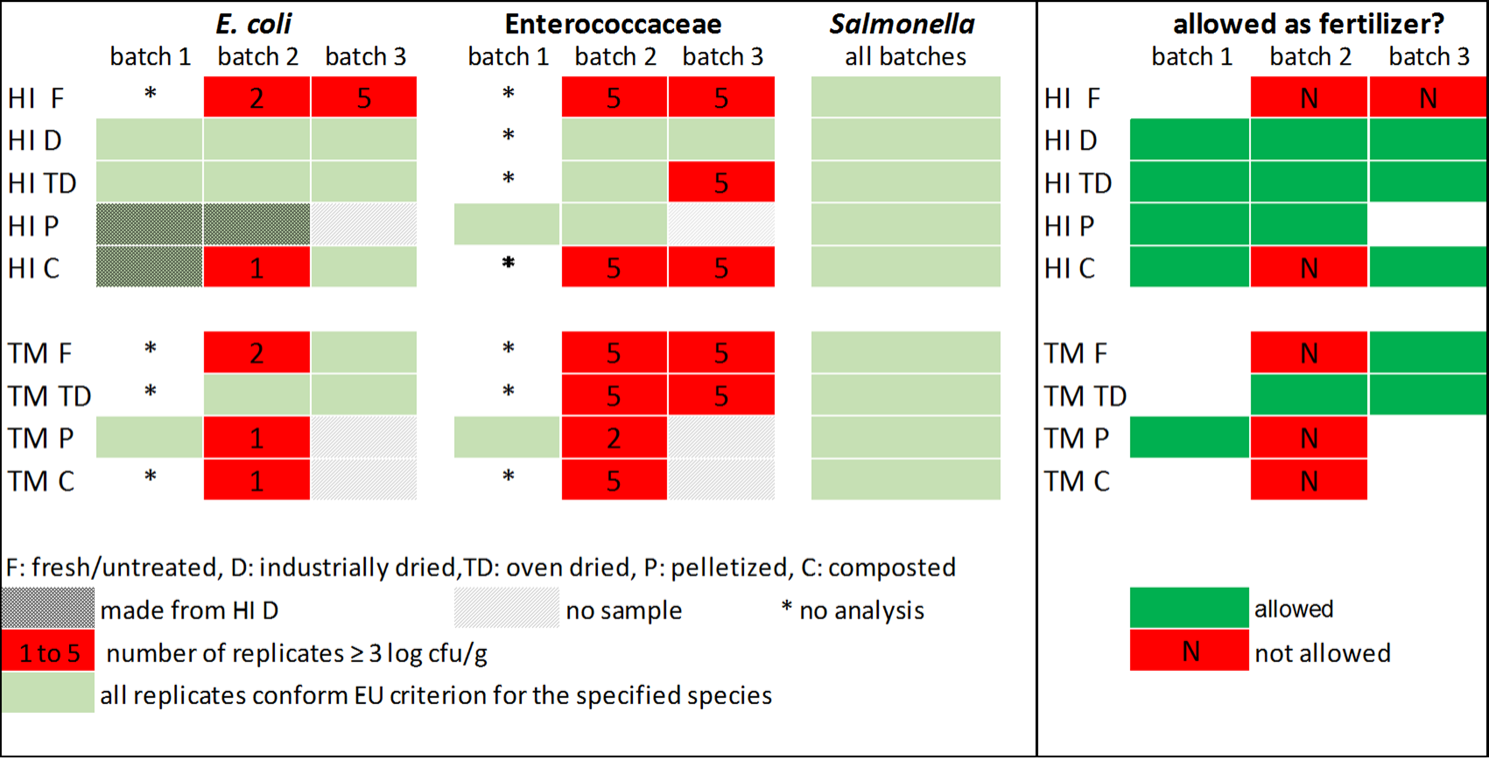
Compliance of tested frass samples from *H. illucens* (HI) and *T. molitor* (TM) rearing with microbiological standards applying to the placing on the EU market as fertilizer and soil improver (Regulation (EU) No 2021/1925)

Regarding compliance of tested frass samples with EU standards for marketing as fertilizer/soil improver, an overview is presented in Fig. 2. Whether or not a frass sample complies with the imposed microbiological criteria seems to depend solely on the number of *E. coli* present. *Salmonella* is not detected in untreated insect frass and no external contamination occurs during processing. *E. coli* are not only present in smaller numbers compared to Enteroccocaceae and are also more sensitive to heat treatment. Even though in processed frass the number of Enterococcaceae often remains above the legal criterion of 3 log cfu/g, the ‘OR’ in legislation renders these numbers irrelevant. This is visualized in Fig. 2, where the right diagram, showing frass conformity with EU standards (allowed, green; not allowed, red), is the spitting image of the most left one, depicting *E. coli* conformity.

## Conclusions

To treat or not to treat? Current EU legislation imposes a heat treatment for frass from insect rearing, aimed at securing its microbiological safety, before it can be marketed as fertilizer in agriculture. However, classification of frass as ‘animal manure’ may not be fully appropriate. Total presence of viable cells and endospores may be comparable to presence in other animal manures, but prevalence of enteric species is lower. To our knowledge, detectable numbers of *Salmonella* spp. in insect frass, have not been reported and generally lower numbers of *E. coli* are found. The imposed processing may therefore be too strict or even unnecessary, increase production costs and impact the frass valorization potential. Considering physical thermal drying, applying heat to black soldier fly larvae and yellow mealworm frass (70 °C for one hour) generates a product compliant with EU legislation but the process is expensive and treatment at lower temperature/for shorter time may achieve a similar result. Pelleting is suited for YM frass given its low moisture content and low *E. coli* counts but BSFL frass needs a prior drying step. Biothermal drying (composting) of BSFL frass will only result in a safe product if generated process temperatures induce sufficient *E. coli* reduction. Composting is however not suited for YM frass hygienization as the process augments the frass water activity and renders it microbiologically more unstable/unsafe than the untreated frass. Independent of the treatment applied, if proper hygiene conditions are met during processing the risk of secondary *Salmonella* contamination can be avoided.

Whether or not either one of these treatments generates frass that complies with the microbiological safety standards depends solely on the number of *E. coli* present. *Salmonella* is hardly ever found in untreated insect frass. *E. coli,* present in smaller numbers compared to Enterococcaceae, is more sensitive to heat and thus more easily reduced. The question may therefore not be ‘To treat or not to treat?’ but ‘When and how to treat?’ and ‘Why *E. coli* OR Enterococcaceae?’.

## Supplementary Information

Additional file 1.

## Data Availability

Data supporting this study are openly available from Lirias (Data Repository of KU Leuven University) at 10.48804/VWRJVW.

## Abbreviations

BSF: Black soldier fly
BSFL: Black soldier fly larvae
YM: Yellow mealworm(s)
HI: *Hermetia illucens*
TM: *Tenebrio molitor*
TVC: Total viable count
PPS: Physiological salt solution
a_w_: Water activity
cfu: Colony forming units
D: Industrial drying
TD: Thermal oven drying
C: Composting, compost
P: Pelleting, pellets
PCA: Plate count agar
VRGB: Violet red bile glucose agar
KAA: Kanamycin esculin azide agar
TBX: Trypton bile-x glucuronide agar
dm: Dry matter
