# Supplementary material for: To treat or not to treat? Impact of hygienization on the microbiological safety of frass from black soldier fly larvae and yellow mealworm production aimed for fertilizer use
Source: Bioresour Bioprocess. 2026 May 4;13(1):64. doi: 10.1186/s40643-026-01030-7 (PMC13139546; doi:10.1186/s40643-026-01030-7)
Supplement: Supplementary file 1 — Additional file 1. [file 40643_2026_1030_MOESM1_ESM.docx]

Supplementary material


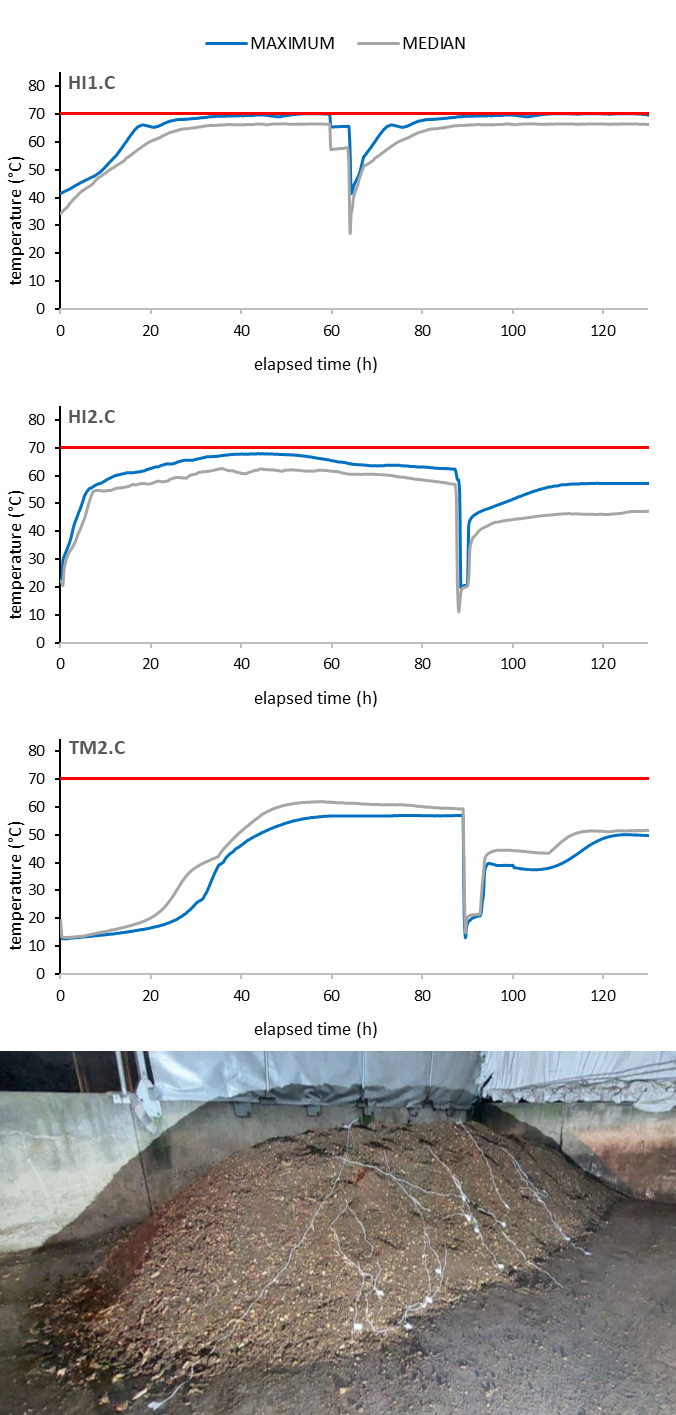


Figure S1: Temperature profiles during heap composting of *H. illucens* (HI) and *T. molitor* (TM) frass. Picture of compost pile with temperature sensors.
